# Construction Notes

**Published:** 1894-09-29

**Authors:** 


### CONSTRUCTION NOTES.

**Stockton Surgical Hospital.**

Owing to the insufficiency of the laundry accommodation of the Stockton Surgical Hospital, the committee have had plans prepared, and a new building is to be fitted up for this purpose. Every modern appliance will be included in this design. The estimated cost of this extension is about £600.

**New Infirmary at Selly Oak Workhouse.**

The Guardians of the King's Norton Union have for a considerable time been occupied with the engrossing question of providing suitable hospital accommodation. They are to be warmly congratulated upon the manner in which they have combined efficiency with economy in the instance presented to us in the construction of this much-needed institution. Designs for the construction of this infirmary were accepted from Mr. Daniel Arkell, of King's Norton. In the centre of the building is the administrative block, connected on either side by a wide corridor to two handsome pavilions. These latter contain the sick wards for males and females. By this arrangement the sexes are kept apart. The building is designed with the intention of working it at a future date as a self-contained and separate establishment, but for the present it will be worked in conjunction with the workhouse. The ward arrangement furnishes eight large rooms, containing 96 beds for males and the same number for females; eight smaller wards (for special cases), to contain 16 beds for males and 16 for females; and eight smaller one-bed wards for extreme cases; two lying-in wards with 10 beds, and two labour wards adjacent. There is thus total accommodation afforded for 250 persons. All the wards are provided with fireproof exit staircases in case of emergency. The bath and lavatory apparatus is on a most complete scale. Great care and consideration has been bestowed upon the provision of a nurses duty-room, with pantry and linen store adjoining. Lifts have been included for conveying patients from ground to first-floor beds. Receiving wards for males and females are set aside for incoming patients, with bath-room and lavatory accommodation to each. The administrative block, near the front entrance, contains matron's room, board-room, doctor's private sitting-room, with dispensary next the corridor. Cooking is done at the rear of the centre block. The basement portion of the building is utilised for stores. Varandahs are placed at the end of the sick wards for convalescent patients, in lieu of the day-rooms usually set aside for that purpose. The building and land it is estimated will cost about £30,000.

**Dumfermline Cottage Hospital.**

This hospital, just opened, faces south. The entrance door leads into a spacious outer lobby, separated from the main hall by a wood and glass screen. On the right of the hall is the operating room; this has a tiled floor, the walls being coated with Keane's cement, making it impervious to germs and damp. Sinks and basins are also fitted up, and every accommodation necessary for a perfect operating theatre has been introduced. Wide and airy corridors lead east and west to the male and female wards respectively. Each of these wards contain six beds, the floor and cubic space fulfilling all necessary conditions in regard to all requirements of health. Opening off each ward is a separate wing containing baths, sinks, etc. The corners of the wards are carefully rounded off, and an absence of cornice and projections alike prevent any accumulation of dust. Outside these wards and facing south is a large rail-lined balcony, on which convalescent patients can sit and enjoy the air. Between are placed the matron's and nurses' rooms, the doctors' and committee rooms. The central portion of the building is carried up another storey, and in this upper floor are two wards of two beds each for special cases, two bathrooms, water closets, napery room, night nurses' room, and servants' rooms. Great attention has been paid to the heating and ventilation, the former being through low pressure hot-water apparatus. Hot-water coils of a pattern specially constructed for the architects are placed in the windows, and fresh air is admitted through them. Trenches for fresh air lead from the wards and other parts of the building into the central ventilator. This steam coil is heated by a separate furnace, so that it can be used in summer when the large pressure is not required. A well equipped laundry and a small mortuary are erected at the north-east corner of the site. The grounds surrounding the hospital are nicely laid out. The total cost of the building is estimated at about £4,500. The architect of the building is Mr. Sydney Mitchell, of Messrs. Mitchell and Wilson, Edinburgh; the contractors for the mason work being James Stewart, of Dumfermline; healing and ventilation, Mackenzie and Moncur, of Edinburgh.

